# Assessment of Domestic Goats as Models for Experimental and Natural Infection with the North American Isolate of *Rickettsia slovaca*

**DOI:** 10.1371/journal.pone.0165007

**Published:** 2016-10-14

**Authors:** Nicole Lukovsky-Akhsanov, M. Kelly Keating, Pamela Spivey, George W. Lathrop, Nathaniel Powell, Michael L. Levin

**Affiliations:** 1 Comparative Medicine Branch, Division of Scientific Resources, National Center for Emerging and Zoonotic Infectious Diseases, Centers for Disease Control and Prevention, Atlanta, Georgia, United States of America; 2 *Rickettsial Zoonoses Branch*, Division of Viral and Rickettsial Diseases, National Center for Infectious Diseases, Centers for Disease Control and Prevention, Atlanta, Georgia, United States of America; 3 Infectious Diseases Pathology Branch, Division of High-Consequence Pathogens and Pathology, National Center for Emerging and Zoonotic Infectious Diseases, Centers for Disease Control and Prevention, Atlanta, Georgia, United States of America; University of Minnesota, UNITED STATES

## Abstract

*Rickettsia slovaca* is a tick-borne human pathogen that is associated with scalp eschars and neck lymphadenopathy known as tick-borne lymphadenopathy (TIBOLA) or *Dermacentor*-borne necrosis erythema and lymphadenopathy (DEBONEL). Originally, *R*. *slovaca* was described in Eastern Europe, but since recognition of its pathogenicity, human cases have been reported throughout Europe. European vertebrate reservoirs of *R*. *slovaca* remain unknown, but feral swine and domestic goats have been found infected or seropositive for this pathogen. Recently, a rickettsial pathogen identical to *R*. *slovaca* was identified in, and isolated from, the American dog tick, *Dermacentor variabilis*. In previous experimental studies, this organism was found infectious to guinea pigs and transovarially transmissible in ticks. In this study, domestic goats (*Capra hircus*) were experimentally inoculated with the North American isolate of this *R*. *slovaca*-like agent to assess their reservoir competence–the ability to acquire the pathogens and maintain transmission between infected and uninfected ticks. Goats were susceptible to infection as demonstrated by detection of the pathogen in skin biopsies and multiple internal tissues, but the only clinical sign of illness was transient fever noted in three out of four goats, and reactive lymphoid hyperplasia. On average, less than 5% of uninfected ticks acquired the pathogen while feeding upon infected goats. Although domestic goats are susceptible to the newly described North American isolate of *R*. *slovaca*, they are likely to play a minor role in the natural transmission cycle of this pathogen. Our results suggest that goats do not propagate the North American isolate of *R*. *slovaca* in peridomestic environments and clinical diagnosis of infection could be difficult due to the brevity and mildness of clinical signs. Further research is needed to elucidate the natural transmission cycle of *R*. *slovaca* both in Europe and North America, as well as to identify a more suitable laboratory model.

## Introduction

*Rickettsia slovaca* is a tick-borne human pathogen, which is often associated with scalp eschars and neck lymphadenopathy. It was originally discovered in the *Dermacentor marginatus* tick collected in central Slovakia in 1968 and further identified in the *Dermacentor reticulatus* tick throughout Europe [1,2,3, 4]. In 1997, this agent was for the first time associated with a human case in France where an infection resulted in fever, arthralgia, fatigue, severe headache, necrotic eschar, enlarged occipital lymph nodes [3]. Since then, human cases due to *R*. *slovaca* infection have been reported throughout Europe [2,5]. The seasonal peak of human cases occurs in the winter and spring months coinciding with increased levels of *D*. *marginatus* activity, providing further evidence of maintenance of the organism in this tick vector [2]. Over 20% of *D*. *marginatus* ticks collected from animals and from vegetation in western and central Slovakia in 2004–2010 were positive for *R*. *slovaca*. This highlights a risk of humans contracting disease and the need for further investigative research [6].

Typical disease course in human patients includes enlarged lymph nodes, eschar, fever, rash and alopecia in addition to possible neurological symptoms. The syndrome is variably and interchangeably described as tick-borne lymphadenopathy (TIBOLA) or *Dermacentor*-borne necrosis erythema and lymphadenopathy (DEBONEL) or scalp eschar and neck lymphadenopathy after tick bite (SENLAT) due to common attachment of the tick vector to the scalp [7]. It is important to note that other rickettsial agents (*Candidatus* Rickettsia rioja and *R*. *raoultti*) have also been implicated in causing overt clinical disease manifested as TIBOLA/DEBONEL. These closely related spotted fever group (SFG) *Rickettsia* spp. may be difficult to distinguish except molecularly.

Recently, a rickettsial pathogen genetically identical to *R*. *slovaca* has been identified in and isolated from the American dog tick, *Dermacentor variabilis* [8]. This new isolate has been shown to be pathogenic in guinea pigs and is transovarially transmissible in *D*. *variabilis* for at least 3 generations [9].

Natural cycles of tick-borne diseases are complex. In addition to the pathogen and its arthropod vector(s), they include vertebrate reservoirs, which sustain ticks by serving as a source of blood-meals while also providing a route for transmission of the pathogen between infected and uninfected ticks, thus amplifying the prevalence of infection within the vector population. Therefore, identification of vertebrate reservoirs is a vital part of our understanding of natural cycles of tick-borne diseases. Likewise, laboratory studies in transmission, maintenance, infectivity, virulence, and pathogenicity of tick-borne agents require both live vectors (ticks) and appropriate animal models to reproduce a natural route of infection via a tick bite [10].

In Europe, wild boars are regarded as preferred natural hosts of *D*. *marginatus*, however, adult ticks routinely feed on domestic animals including small ruminants and cattle. In North America, immature *D*. *variabilis* infest a variety of small and medium-size mammals, while adult ticks feed on different species of carnivorans (order Carnivora) including domestic dogs, wolves, foxes, jackals, coyotes, raccoons and others. Similarly to the European vectors of *R*. *slovaca*, *D*. *variabilis* adults also frequently feed on domestic ungulates including goats [11]. This raises a possibility that some of the domestic animals may be involved in maintenance of the tick-transmitted *R*. *slovaca* in peridomestic cycles [5]. In the Catalonia region of Spain, for example, three out of 91 tested domestic goats (3.3%) had elevated antibody titers (>1:320) against *R*. *slovaca*. Although detection of antibodies reactive with one of SFG *Rickettsia* spp. is difficult to interpret due to the wide cross-reactivity between group members, presence of *R*. *slovaca* DNA had been demonstrated in the blood of one goat with an anti-*Rickettsia* antibody titer of 1:160 [12]. These data suggest that domestic goats may be exposed to the pathogen and even become infected.

Nevertheless, neither the susceptibility of domestic goats to *R*. *slovaca*, nor their capacity for amplification of the pathogen and its transmission between infected and uninfected ticks are known. Clinical, pathological, and immunological progression of *R*. *slovaca* infection in these animals has also not been described. This study aims to provide clarification on disease pathogenesis in domestic goats infected with the North American isolate of *R*. *slovaca* as well as their ability to transmit the pathogen to uninfected ticks and to establish or maintain a natural transmission cycle. We hypothesized that if domestic goats are susceptible to *R*. *slovaca*, they would develop discernable clinical signs of infection, transmit the pathogen to uninfected ticks, and be suitable for use as models in studies of natural transmission and proliferation of this rickettsial species.

## Materials and Methods

The Centers for Disease Control and Prevention animal care and use program and vivarium are accredited by the Association for the Assessment and Accreditation of Laboratory Animal Care International (AAALAC). Housing, husbandry, and procedures for this study were performed in accordance with the Guide for the care and Use of Laboratory Animals [13]. The four goats used in this study were originally sourced from a United States Department of Agriculture-Veterinary Services accredited farm and had annual physicals including full bloodwork including Caprine Arthritis-Encephalitis virus (CAE) titers and deworming performed. They were previously (in 2012–2013) exposed to and subsequently cleared of *Anaplasma phagocytophilum* infection. Between the past and the present study, goats were kept outdoors on a fenced, tick-free pasture. For the duration of the current study, which was approved by the Institutional Animal Care and Use Committee (IACUC) at the Centers for Disease Control and Prevention (CDC), four adult female mixed breed goats were singly housed under BSL-2 containment in an indoor facility, which precluded unintended exposure to any arthropod-borne agent including *Rickettsia* spp. The absence of antibodies to spotted fever group (SFG) rickettsiae in each goat was confirmed prior to enrollment into the study by the indirect immunofluorescence assay (IFA) as described below. Baseline assessment of the complete blood-cell counts and blood chemistry was conducted three days after relocation of the goats indoors and three days prior to initiation of the study. Food and water were provided ad libitum. The appetite, behavior, disposition, and level of activity of each goat were monitored daily throughout the study. The body temperature of each goat was measured every morning; and temperatures ≥39.5°C were defined as febrile. Venous blood and serum samples were collected three times per week for the differential blood cell counts, serum chemistry panels, and PCR.

A pathogen-free colony of *D*. *variabilis* was established in 1999 from wild adult ticks collected in the vicinity of Atlanta, GA. Ticks are maintained in the Medical Entomology Laboratory at Centers for Disease Control (CDC) under standard laboratory conditions as described previously [10]. Naïve New Zealand white rabbits (*Oryctolagus cuniculus*) were used as hosts for all developmental stages of uninfected ticks. A separate colony of *D*. *variabilis* ticks infected with *R*. *slovaca* was established in 2012 as previously described [9].

Two goats (#29 and #64) were randomly selected for needle inoculation with *R*. *slovaca* and the other two goats (#33 and #44) were fed upon by infected adult *D*. *variabilis* ticks. Each needle-inoculated goat received an infectious dose containing 1x10^6^ DNA copies of *R*. *slovaca* isolate propagated in Vero cell culture and suspended in 4ml of PBS immediately prior to inoculation. Each goat received 2 ml of the inoculum intravenously (jugular vein) and 2 ml—subcutaneously between the shoulder blades. In goats #33 and #44 exposed to *R*. *slovaca* via tick bite, skin between shoulder blades was shaved and a piece of tubular stockinet was adhered to the skin using skin glue forming a “feeding bag”. Twenty male and twenty female adult *D*. *variabilis* ticks from the *R*. *slovaca*-infected colony were placed inside each feeding bag (for containment) and allowed to feed to repletion. Animals were fitted with Elizabethan collars and/or safety jackets (www.SafetyPUPXD.com) to prevent damage to enclosed ticks due to normal behavior of the goats such as rubbing against enclosure. Uninfected larval *D*. *variabilis* ticks were placed on all four goats one day after infection–either via needle inoculations or via tick bites–for acquisition feeding. Feeding bags were checked daily for the duration of infestation (7–10 days) and engorged ticks were removed upon detachment from the host. Additionally, serial 2mm skin punch biopsies were collected once a week at the tick attachment sites—between the shoulder blades and a distant site located at/near the hip to evaluate for tropism of the organism in skin tissues.

One each of the needle-inoculated (#64) and tick-inoculated (#44) goats were euthanized 22 days post inoculation (DPI) and subjected to necropsy and pathology examinations. Animals selected for necropsy were sedated with ketamine followed by pentobarbital intravenously for humane euthanasia. Full necropsy was performed and samples of major organs, including skin, liver, spleen, kidney, lung, heart, lymph nodes, bone marrow from femur and cerebrospinal fluid were collected for histopathology, immunohistochemical and PCR analyses. The two remaining goats were monitored until 48 DPI, then treated with injectable oxytetracycline (200 mg/kg intramuscularly every 48 hours for a total of three treatments) and returned to the pasture.

Serum samples were stored at -70°C until tested using Indirect Immunofluorescence Assay (IFA). IFA was performed, as previously described [14] to detect antibodies to spotted fever group (SFG) *Rickettsia* using FITC-labeled goat α-rabbit IgG (H+L) conjugate (KPL Inc., Gaithersburg, MD) diluted per manufacturer’s instructions and whole cell *Rickettsia slovaca* antigen. Sera were initially screened at 1/16 dilution and positive samples were titrated to endpoint in a two-fold dilution series.

Samples of whole blood, skin biopsies, internal tissues collected during necropsy, as well as representative samples of engorged and molted ticks, were tested by PCR. Tick DNA was extracted using the DNeasy Blood and Tissue kit (Qiagen Inc., Valencia, CA) according to the manufacturer’s protocols and eluted in 100ul of buffer (final volume). Whole blood DNA was extracted using the Qiagen FlexiGene DNA extraction kit (Qiagen Inc., Valencia, CA) according to the manufacturer`s protocol and eluted in 100uL of elution buffer. Tissue DNA extraction and the PCR procedure for detection of rickettsial DNA were the same as those for testing tick DNA samples. Real-time PCR was used to detect the *omp*A gene of SFG *Rickettsia* as described previously [15]. A plasmid of *R*. *massiliae* and distilled water were used as a positive and negative controls, respectively, and included in each PCR run.

A complete necropsy was performed at the time of euthanasia. Tissue samples were fixed in 10% buffered formalin, paraffin-embedded and sectioned at 4 μm for staining with routine hematoxylin-eosin. Immunohistochemistry using an immunoalkaline phosphatase was performed on select blocks. The primary antibody used was a polyclonal rabbit antibody raised against *R*. *rickettsia* that is known to cross-react with other SFG *Rickettsia* species as previously described [16]. *R*. *slovaca*-infected Vero cells were used as positive controls, and normal rabbit serum in place of primary antibodies were used as a negative control.

Standard errors were calculated to assess the frequency of pathogen detection by PCR and the Chi-squared test was used to evaluate the statistical significance of differences between results of this and previously published studies.

## Results

**Fig 1:**
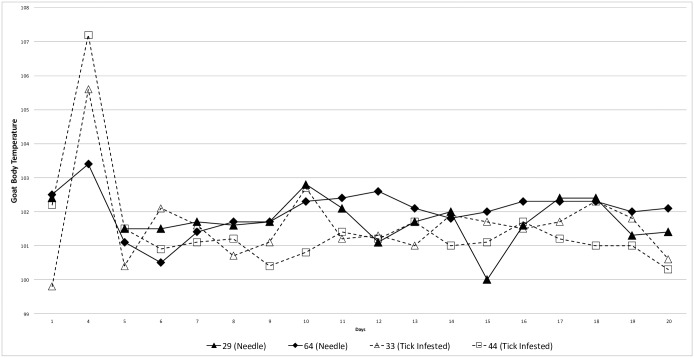
Core body temperature in goats infected with the North American Isolate of *Rickettsia slovaca*.

Both goats exposed to *R*. *slovaca*-infected ticks (#33 and #44) developed fever at 4 DPI with core temperatures rising to 40.9°C and 41.8°C respectively (Fig 1). On the same day, one of the needle-inoculated goats (#64) also became borderline febrile (39.7°C). Animals appeared subdued during the febrile period although physical examinations resulted in no other significant findings. All goats defervesced within the next 24 hours and body temperatures remained within the normal range for the remainder of the observation period. Goat #29 in the needle-inoculated group developed submandibular pitting edema 6 DPI extending the cranial 1/5^th^ portion of the neck. The edema did not appear painful nor did it impede eating or drinking. It resolved within 3 days without clinical intervention. No other abnormalities were noted in any of the four goats. Complete blood count and chemistry values for baseline and post-inoculation samples were variable between samples and between goats, but all remained within normal ranges for the species with no trends noted.

Adult ticks successfully fed on goats #33 and #44; all engorged females and all male ticks were removed from the feeding bags within 10 days after placement. 38 ticks (19 males and 19 females) were tested for the presence of rickettsial DNA and all 38 (100%) were found to be PCR-positive. Uninfected *D*. *variabilis* larvae completed their engorgement on goats within 4–5 days after placement– 5–6DPI. In the cohort of engorged larvae that were fed upon goats #33 and #44 together with infected adult ticks, 20 out of 25 (80.0±8.2%) contained rickettsial DNA. When these ticks molted into nymphs, only 2 out of 33 (6.1±4.2%) remained PCR-positive. A similar drop in the prevalence of infection was noted among ticks acquisition-fed upon needle-inoculated goats. Among engorged larvae, 9 out of 50 tested ticks (18.0±5.5%) contained rickettsial DNA, whereas only 2 out of 50 tested ticks (4.0±2.8%) tested PCR-positive among freshly molted nymphs. Overall, goats transmitted the pathogen to 4.8±2.4% of nymphs that were acquisition-fed as larvae during the height of the fever response in animals.

Rickettsial DNA was detected in the skin samples taken from both sites of tick feeding–between the shoulders and from the hip. In goats exposed to infected adult ticks, biopsies from both locations were PCR-positive by 8 DPI. In needle-inoculated goats, only one (hip site) out of 4 samples were positive on 8 DPI, but rickettsial DNA was detectable at both sites by 14 DPI (Table 1)Meanwhile, none of the 46 blood samples taken from the four goats between 4 and 22 DPI were positive. Rickettsial DNA was detected in multiple organ tissues collected from two goats during necropsy at 22 DPI. In the needle-inoculated goat #64, rickettsial DNA was present in liver, spleen, kidney and bone marrow. In the tick-inoculated goat # 44, samples of spleen, lung, and cerebrospinal fluid (CSF) were PCR-positive. Overall, 7 out of 15 (46.6±13.3%) tissue samples collected from the two goats during necropsy tested PCR-positive for *R*. *slovaca*.

**Table 1:** Detection of *Rickettsia slovaca* DNA in the skin of goats following inoculation via needle or tick bite.

DPI	#29 (needle)		#64 (needle)		#33 (tick)		#44 (tick)	
	Tick site*	Hip	Tick site	Hip	Tick site	Hip	Tick site	Hip
4	Nd**	-	Nd**	-	Nd**	-	Nd**	-
8	-	-	-	+	+	+	-	-
14	+	-	+	+	+	+	+	+
22	+	-	+	+	+	-	+	+

* The site of attachment of infected *Dermacentor variabilis* adults and/or acquisition-fed larvae–between the shoulder blades.

**Not done

Histologic evaluation of organs collected during necropsy at 22 DPI revealed reactive and hyperplastic changes of lymphoid tissue (thymus, bronchus associated lymphoid tissue, spleen) and chronic eosinophilic and lymphoplasmacytic perivascular dermatitis with dermal fibrosis in the areas of tick feeding or previous biopsy. A mild patchy eosinophilic pneumonia was present and secondary to lungworm infection. There was no immunohistochemical evidence of *R*. *slovaca* in any tissues, including skin, lymph nodes, liver, spleen, heart, thymus, lung, or kidney.

## Discussion

Previous studies demonstrated that European wild boars and domestic goats may be susceptible to *R*. *slovaca* infection [12,17,18,19], and that occupational or habitual contact with wild or domestic animals is associated with increased risk of exposure to *R*. *slovaca* in humans [20]. Our study was initiated to assess the suitability of domestic goats as animal models of infection with the North American isolate of *R*. *slovaca* and their ability to maintain or propagate the natural transmission cycle of this pathogen in the peridomestic environment. Following exposure to the isolate [21] via either needle-inoculation or infestation with infected *D*. *variabilis* ticks, rickettsial DNA was routinely detected in the skin biopsies and in internal organs of goats euthanized at 22 days after exposure. This indicates that goats are susceptible to infection with the North American isolate of *R*. *slovaca*. Of note, rickettsial DNA was detected in the skin samples not only at the site of tick attachment between the shoulder blades, but also in biopsies taken from the hip as early as 8–14 days after needle inoculation, indicating both that generalized infection was established and that the pathogen relatively quickly disseminated throughout the skin of the animal.

However, this infection appeared to be mild and self-limiting as the only clinical sign of illness was a short spike in body temperature observed in three out of four goats at 4 DPI. Submandibular edema recorded in one of the four goats could be due to repeated venipuncture-causing temporary localized tissue inflammation or e-collar placement and could not be unequivocally attributed to the specific rickettsial infection. As expected, eschars were found at the site of tick feeding, but no other clinical signs were observed and comprehensive blood analysis did not detect any consistent changes. Histopathological changes were nonspecific and secondary to generalized immune stimulation (lymphoid hyperplasia) or to surface trauma from arthropod bite or a previous biopsy procedure with no direct evidence of active rickettsial infection, which is different from observations made recently in guinea pigs where rickettsiae-associated lesions were observed in three out of three needle-inoculated animals[9]. This difference may be related to the different timing of euthanasia between the two studies, or more likely the differences in animal models, including species permissiveness to infection, route of infection, and relative amount of inoculum. Whereas pathology in guinea pigs was assessed at 2 weeks post inoculation, goats in our study were euthanized 3 weeks after exposure. An additional week could have allowed for recovery and healing of any damages inflicted by infection. This hypothesis is supported by persistence of rickettsial DNA in internal tissues at 22 DPI, detection by PCR and not immunohistochemistry suggests low rickettsial burden, which could be compatible with resolving infection.

Interestingly, no rickettsial DNA was detected in blood samples collected during the first 3 weeks after introduction of the pathogen. These results contrast the previously reported detection of *R*. *slovaca* DNA in the blood of a goat in Spain despite the presence of antirickettsial antibodies in that animal [12]. They also differ from earlier observation in the guinea pig model infected with the same North American isolate of *R*. *slovaca*, where an average of 6.3±2.7% of 80 blood samples were PCR positive, although pathogen detection in blood was inconsistent and unpredictable [22]. The stated difference between results of this study and the one conducted in guinea pigs is not statistically significant (χ2 = 2.2089; p = 0.08) most likely due to relatively low sample numbers.

Some of *D*. *variabilis* larvae feeding upon the infected goats concurrently with the height of fever were able to acquire the pathogen. When uninfected larvae were fed together with infected adult ticks, 80% of the former became PCR-positive. However, only 6.1±4.2% of resulting nymphs retained rickettsial DNA after molting. This was similar (χ2 = 1.2665; p = 0.26) to the previously reported 11.7±3.7% prevalence of infection in nymphs resulting from co-feeding of infected and uninfected ticks upon guinea pigs [9]. The overall prevalence of infection in nymphal ticks that were acquisition fed upon goats during the height of the fever response was 4.8±2.4%. Considering that goats naturally carry relatively few immature *D*. *variabilis*, it is unlikely they would produce significant numbers of ticks infected with the North American isolate of *R*. *slovaca* via horizontal transmission.

## Conclusion

Domestic goats are susceptible to the newly described North American isolate of *R*. *slovaca* when the pathogen is introduced via either needle-inoculation or by bite of the infected tick. However, the resulting infection is mild and self-limiting with no obvious clinical or hematological signs other than a short spike in body temperature. This makes the domestic goat *Capra hircus* a poor model for clinical and veterinary studies of this agent. Feeding of uninfected ticks upon *R*. *slovaca*-infected goats resulted in relatively low prevalence of infection. Therefore domestic goats are likely to play only a minor role in the natural transmission cycle of this pathogen. Our results suggest that goats do not propagate the North American isolate of *R*. *slovaca* in peridomestic environments and clinical diagnosis of infection could be difficult to recognize. Further research is needed to elucidate the natural transmission cycle of *R*. *slovaca* both in Europe and North America, as well as to identify a more suitable laboratory model.

## Abbreviations

TIBOLA: tick-borne lymphadenopathy
DEBONEL: *Dermacentor*-borne necrosis erythema and lymphadenopathy
SENLAT: scalp eschar and neck lymphadenopathy after tick bite
CAE: Caprine Arthritis-Encephalitis virus
DPI: Days post infection
SFG: Spotted fever group
IFA: Indirect Immunofluorescence Assay
CDC: Centers for Disease Control and Prevention
